# PAPST, a User Friendly and Powerful Java Platform for ChIP-Seq Peak Co-Localization Analysis and Beyond

**DOI:** 10.1371/journal.pone.0127285

**Published:** 2015-05-13

**Authors:** Paul W. Bible, Yuka Kanno, Lai Wei, Stephen R. Brooks, John J. O’Shea, Maria I. Morasso, Rasiah Loganantharaj, Hong-Wei Sun

**Affiliations:** 1 Laboratory of Skin Biology, Intramural Research Program, National Institute of Arthritis and Musculoskeletal and Skin Diseases, Bethesda, Maryland, United States of America; 2 Molecular Immunology and Inflammation Branch, Intramural Research Program, National Institute of Arthritis and Musculoskeletal and Skin Diseases, Bethesda, Maryland, United States of America; 3 State Key Laboratory of Ophthalmology, Zhongshan Ophthalmic Center, Sun Yat-sen University, Guangzhou, China; 4 Biodata Mining and Discovery Section, Office of Science and Technology, Intramural Research Program, National Institute of Arthritis and Musculoskeletal and Skin Diseases, Bethesda, Maryland, United States of America; 5 Laboratory of Bioinformatics, Center for Advanced Computer Studies, University of Louisiana at Lafayette, Lafayette, Louisiana, United States of America; King Abdullah University of Science and Technology, SAUDI ARABIA

## Abstract

Comparative co-localization analysis of transcription factors (TFs) and epigenetic marks (EMs) in specific biological contexts is one of the most critical areas of ChIP-Seq data analysis beyond peak calling. Yet there is a significant lack of user-friendly and powerful tools geared towards co-localization analysis based exploratory research. Most tools currently used for co-localization analysis are command line only and require extensive installation procedures and Linux expertise. Online tools partially address the usability issues of command line tools, but slow response times and few customization features make them unsuitable for rapid data-driven interactive exploratory research. We have developed PAPST: Peak Assignment and Profile Search Tool, a user-friendly yet powerful platform with a unique design, which integrates both gene-centric and peak-centric co-localization analysis into a single package. Most of PAPST’s functions can be completed in less than five seconds, allowing quick cycles of data-driven hypothesis generation and testing. With PAPST, a researcher with or without computational expertise can perform sophisticated co-localization pattern analysis of multiple TFs and EMs, either against all known genes or a set of genomic regions obtained from public repositories or prior analysis. PAPST is a versatile, efficient, and customizable tool for genome-wide data-driven exploratory research. Creatively used, PAPST can be quickly applied to any genomic data analysis that involves a comparison of two or more sets of genomic coordinate intervals, making it a powerful tool for a wide range of exploratory genomic research. We first present PAPST’s general purpose features then apply it to several public ChIP-Seq data sets to demonstrate its rapid execution and potential for cutting-edge research with a case study in enhancer analysis. To our knowledge, PAPST is the first software of its kind to provide efficient and sophisticated post peak-calling ChIP-Seq data analysis as an easy-to-use interactive application. PAPST is available at https://github.com/paulbible/papst and is a public domain work.

## Introduction

ChIP-Seq, one of the most powerful research applications of Next Generation Sequencing (NGS) technology, is fundamental to the investigation of TF-to-DNA interactions and histone modifications. A general ChIP-Seq data analysis pipeline includes a) mapping sequence reads to a genome, b) calling statistically significant peaks, and c) broad down-stream analysis of called peaks including co-localization analysis and assignment of peaks to genes and other annotated genomic regions. There are well-established mapping programs [1–3] and a large number of peak-calling programs [4–8] including specialized peak-callers with robust normalization features [9]. However, availability of easy-to-use yet powerful platforms for post peak-calling data analysis has been limited. Addressing this need is particularly important for bench scientists who, in most cases, are responsible for making biological sense of the data. Chojnowski et al. [10] has recently highlighted the lack of packages for post peak-calling analysis and the need for more user-friendly software to empower a greater number of researchers in the community. Their program, jChIP, is the only downstream ChIP analysis package with a cross platform graphical interface. That program focuses on visualization of raw data as histograms and viewing quality check information. Two other recently developed tools, with some co-localization analysis features, include PAVIS [11] and ChIPseek [12]. Both are web-based. The former is peak-centric only, and the latter brings online some features of popular Linux command line tools such as HOMER [13] and BEDTools [14]. Online tools such as these have the advantage of few system requirements and support more devices. However, this advantage comes at the cost of limits placed on the amount of data processed, longer response times from the software, and fewer customization features. In comparison, a stand-alone desktop tool gives the user more customization and control over the data being analyzed, as successfully demonstrated by the popular Java platform genomics viewer IGV [15].

**Fig 1:**
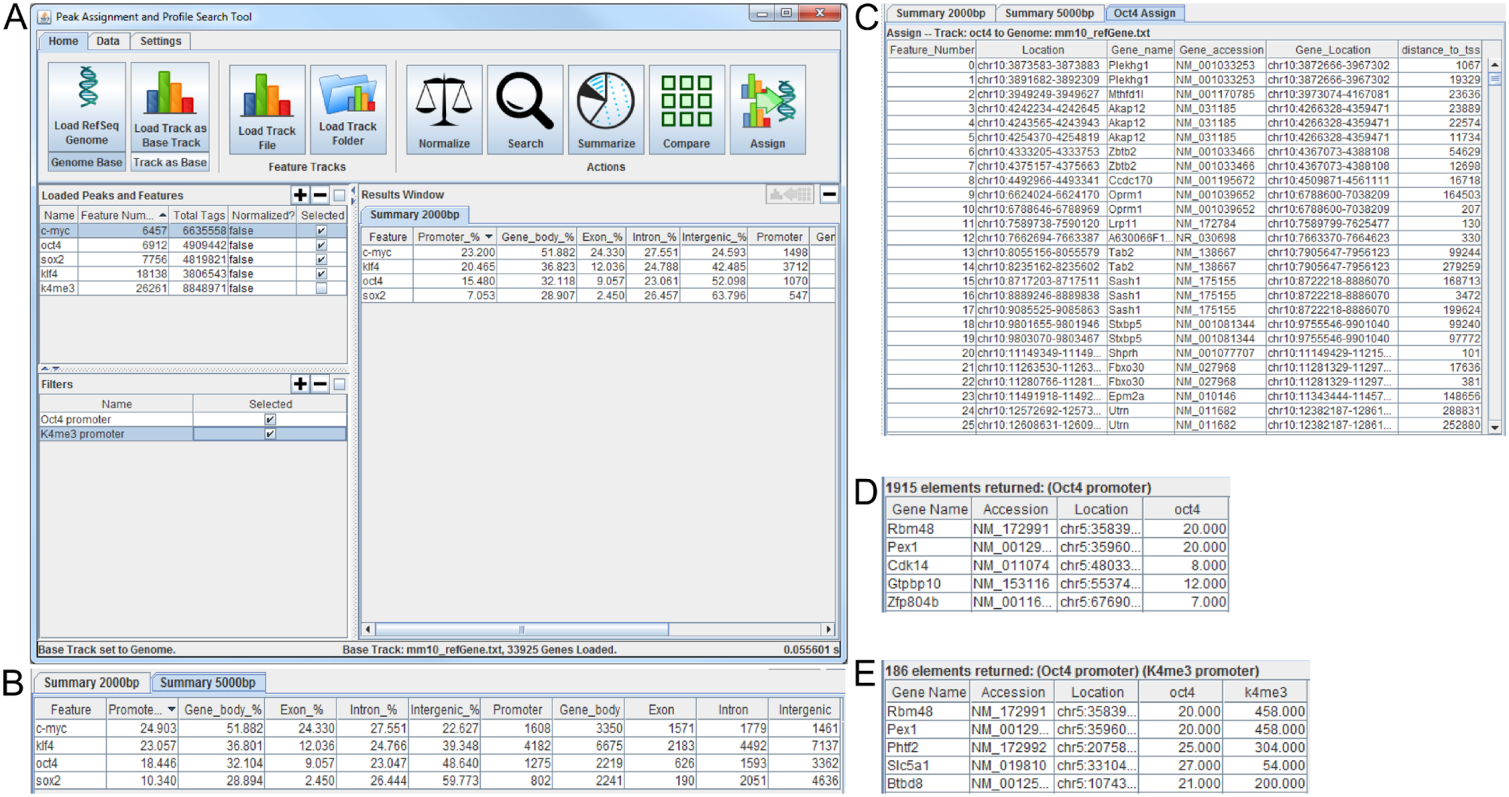
PAPST in action. The graphic design of PAPST was modelled after familiar office productivity software. A. Using the PAPST Summarize command shows the distribution of peaks relative to genomic features. B. Modifying the promoter distance changes the results allowing customization. C. PAPST assigns genes to their nearest TSS. D & E. PAPST searches for genes having a pattern of (D) Oct4 binding in their promoters or (E) K4me3 binding in their promoters and an Oct4 tag value of greater than 20.

We have developed PAPST (peak assignment and profile search tool), with bench scientists in mind, as an easy-to-use yet powerful stand-alone platform for post peak-calling data analysis, focusing on co-localization analysis of transcription factors and histone modifications, a critical component of integrated ChIP-Seq data analysis (Fig 1A). PAPST has been especially designed for efficient data-driven exploratory research. Among its most notable distinct features are its lightning-fast speed, its capability to perform customizable co-localization analysis on an unlimited number of factors, and its unique design that integrates both gene-centric and peak-centric (see the Methods section for Special term definitions) ChIP-Seq data analysis into a single package. These powerful and unique features are highly desirable in data-driven exploratory research, where data analysis parameters can be quickly changed and the output results can easily be used as inputs for the next round of analysis on a fine-tuned hypothesis. We present first examples of the main features of PAPST then apply it to select mouse embryonic stem cell (ESC) ChIP-Seq data [16, 17] and demonstrate its typical applications in post peak-calling ChIP-Seq data analysis, co-localization analysis in particular. The case study presented in this work addresses the analysis of typical and super ESC enhancers ([16] also see the Special term definitions). Specifically, we show PAPST’s rapid applications in a) identifying ESC enhancers as regions co-localized by the master TFs Oct4, Sox2, and Nanog; b) screening for genes with co-promoter-occupancy of the three master TFs and H3K27Ac; c) comparing peak intensities of multiple relevant TFs and EMs in the ESC super enhancers to those in the typical enhancers; d) assigning the ESC super enhancers to key ESC identity genes; and e) performing peak overlap based clustering of 13 TFs. Our PAPST based ESC ChIP-Seq data analysis results, all obtained through a few mouse clicks and within seconds, are highly consistent with those reported in the literature, validating the specific computational approaches implemented in the platform. To our knowledge, PAPST is the first standalone graphical desktop application of its kind for efficient and sophisticated post peak-calling data-driven ChIP-Seq data analysis. PAPST is a cross-platform Java application that is free for any use as part of the public domain. The program package may be downloaded at https://github.com/paulbible/papst, which provides the application jar, a collection of ready to use TF and EM ChIP-Seq data from [17], a comprehensive user guide, and individual tutorials for both PC and Mac users.

## Results

### PAPST performs common downstream ChIP-Seq analysis tasks quickly and interactively

Certain fundamental tasks are common in nature to all post peak-calling downstream ChIP-Seq analysis. Fundamentally, called ChIP-Seq peaks can be represented as a set of genomic coordinates and nearly all common ChIP-Seq analysis methods rely on determining whether peaks overlap or are near to known genomic features (gene-centric) or user-defined genomic features such as peaks from a specific TF experiment (peak-centric). At the heart of PAPST are efficient algorithms (see Methods section) to quickly process a large number of genomic locations to answer distance and overlap queries. The subsections that follow describe PAPST’s solutions to the most general ChIP-Seq analysis tasks. Co-localization analysis, performed by processing the overlaps of multiple sets of significant ChIP-Seq peaks from multiple experiments is an example of one of PAPST’s features presented in the following sections. All the data sets used in this section are available online as part of the tutorial, which presents step by step instructions of example applications. A detailed user guide is also included in the online distribution package of PAPST.

#### Location based distribution analysis

At the global genomic level, the distribution of protein binding with respect to known features provides a fundamental description of binding behavior. PAPST analyzes peak binding in the promoter, gene body, exon, intron, and Intergenic regions quickly with a single mouse click. The summary distribution information is reported as a percentage of total peaks as well as the number of significant peaks that fall into the genomic region. PAPST was used to calculate the binding distribution for four TFs. The result window of PAPST in Fig 1A shows the distributions for Oct4, Sox2, c-Myc, and Klf4 using a 2000 bp promoter. The results using 5000 bp promoter are shown in Fig 1B. Most analysis packages use a one-size-fits-all promoter size while PAPST allows the user to interactively consider multiple settings. Like most features in PAPST, this analysis can be performed simultaneously on multiple sets of ChIP-Seq peaks.

#### Peak assignment to proximal genes

Post peak-calling ChIP-Seq analysis of TFs invariably begins by assigning peaks to the nearest gene transcription start site (TSS) to dissect their behavior. Many TFs drive gene regulation by interacting with the upstream promoter of target genes. Although the current understanding of gene regulation has evolved to include distal TF-to-target gene regulation, assignment to the nearest TSS provides the baseline preliminary analysis that offers insight to TF behavior [16]. This feature is common to nearly all ChIP-Seq analysis packages. PAPST assigns peaks to the nearest gene and displays the results as a sortable table in the results window (Fig 1C). PAPST allows any result table generated to be exported to a spreadsheet format. The complete gene assignment for Oct4 peaks is provided in S1 Table. PAPST also offers batch gene assignment functionality that will calculate the gene assignments for multiple peak sets and place the results into separate files.

#### Robust gene-based search features

Labs studying specific TFs are typically interested in discovering which genes could be direct regulatory targets. PAPST offers customizable search capabilities that allow users to quickly determine which genes have particular TF binding patterns. The powerful filters feature allows users to create sets of criteria for returning genes of interest. A simple but common task would be to find all genes where a specific TF binds in the promoter. To accomplish this, a PAPST user would simply create a filter, select the promoter option (modify the length if desired), and press the search button. The S2 Table contains the search results for genes having Oct4 in their upstream promoters. A sample of these search results is shown in Fig 1D. The genes are placed in a sortable table in the results window allowing the user to prioritize genes based on total tag coverage.

The above example represents the most basic application of the search features. Much more interesting questions are addressed by combining multiple transcription factors and/or histone marks into the search criteria. A natural extension to the promoter search example described above would be to include the activating histone mark H3K4me3 and filter out lower confidence peaks by tag count. To accomplish this a second filter is added to the search that operates on significant peaks derived from an H3K4me3 ChIP-Seq experiment, and the threshold of the Oct4 filter is adjusted to filter out peaks with fewer than 20 reads. PAPST will process all filters and return only genes that meet all the specified criteria (those with both Oct4 and H3K4me3 binding at the promoter and at least 20 reads for the Oct4 peaks). Fig 1E shows a sample of the results returned using these search criteria (the complete list is available in S3 Table). Creative application of an arbitrary number of peaks and filters provides the user with a robust means of specifying complex regulatory and epigenetic patterns to select interesting genes for further analysis.

#### Robust peak and general interval-based search features

This powerful feature, unique to PAPST, generalizes the multiple criteria based search function to any set of genomic regions enabling peak-centric co-localization analysis. In the previous example, a search was constructed to find genes that met multiple criteria across different experiments. Setting the search mode to “Track as Base” enables the selection of a peak set to serve as the genomic locations that are subjected to multiple user-defined filters. This feature allows complex search criteria to be applied to any set of arbitrary genomic regions of interest. As the current understanding of gene regulation evolves, gene-distal regulatory regions will become increasingly important. PAPST provides easy to use tools to explore protein co-localization patterns at any user-defined set of genomic regions. The Example Applications section below describes a case study that utilizes this feature to detect enhancers. Another example of peak-based search features is presented in the tutorial at https://github.com/paulbible/papst.

#### Performance and Usability

Efficient performance and usability are the two main design principles of PAPST. All PAPST’s functions can be selected and executed through a graphical user interface with a few mouse clicks, and the results will return within seconds. In addition, we have also implemented specific features to increase its usability and enhance its ability for efficient data handling. A few examples of PAPST’s usability features are: 1) imbedded customizable parsers simplify importing data from a wide range of file formats, 2) an analysis session of any number of peak sets can be saved into a single file to revisit later or share with a colleague without reloading individual data sets, 3) analysis results can be saved as a csv file for further analysis by spreadsheet programs, 4) specific entries may be easily copied and pasted into the UCSC genome browser for viewing, and 5) analysis results may be quickly reformatted within PAPST and used as a new input for the next round of co-localization analysis, greatly simplifying iterative analyses.

To demonstrate its efficient performance and ease of use, we have timed a complete analysis session in which we loaded Oct4, Sox2, and Nanog peaks, determined regions of co-localized binding, loaded the literature derived normal and super enhancer regions [16], and calculated their agreement with PAPST derived regions in terms of overlapping peaks. PAPST completed the entire 7-step analysis session within 19 seconds (wall clock time), from data loading to result generation. The detailed data are given in Table 1, including the timings for each specific step and the number of mouse clicks needed. We have also tested PAPST, using a typical laptop PC (IBM ThinkPad, 64 bit Windows 7, 8 GB RAM, Intel Core i7 2620M CPU @ 2.70 GHz), on a hypothetical data set of 50 factors (each with 26,261 peaks 1.3 MB each, 65 MB total size) and a co-localization analysis was completed within 1.39 seconds against a peak set (26,261 genomic locations, peak-centric analysis) and 1.54 seconds against RefGene (34150 transcripts, gene-centric analysis), respectively.

**Table 1:** A Complete Analysis Session in PAPST with Timing Information.

Task	Number of mouse clicks	Time
**Load peak files of Oct4, Sox2, and Nanog (23986, 16056, 22463 peaks, respectively)**	**3**	**3 sec**
**Assign Oct4 peaks as the base**	**4**	**4 sec**
**Define search criteria by creating a filter**	**4**	**4 sec**
**Search for regions co-localized by the 3 TFs**	**1**	**1 sec (the search operation itself took 0.051 sec)**
**Reformat the search results into an input file**	**4**	**4 sec**
**Load genomic locations of super-enhancers and typical enhancers**	**2**	**2 sec**
**Calculate overlaps for all 20 pairwise comparisons**	**1**	**1 sec**
**Total**	**19 mouse clicks**	**19 seconds**

PAPST is a very fast and easy to use platform for exploratory research as illustrated by the number of clicks to accomplish a task as well as the time.

### Example Applications of PAPST

The following sections describe case applications of PAPST, with pubic ChIP-Seq datasets, to demonstrate its typical uses and its great potential in cutting-edge research. The results as compared to those published also validate the algorithms and approaches specifically implemented in PAPST.

#### Co-localization analysis to identify enhancers and screen for genes with common TF bindings

**Fig 2:**
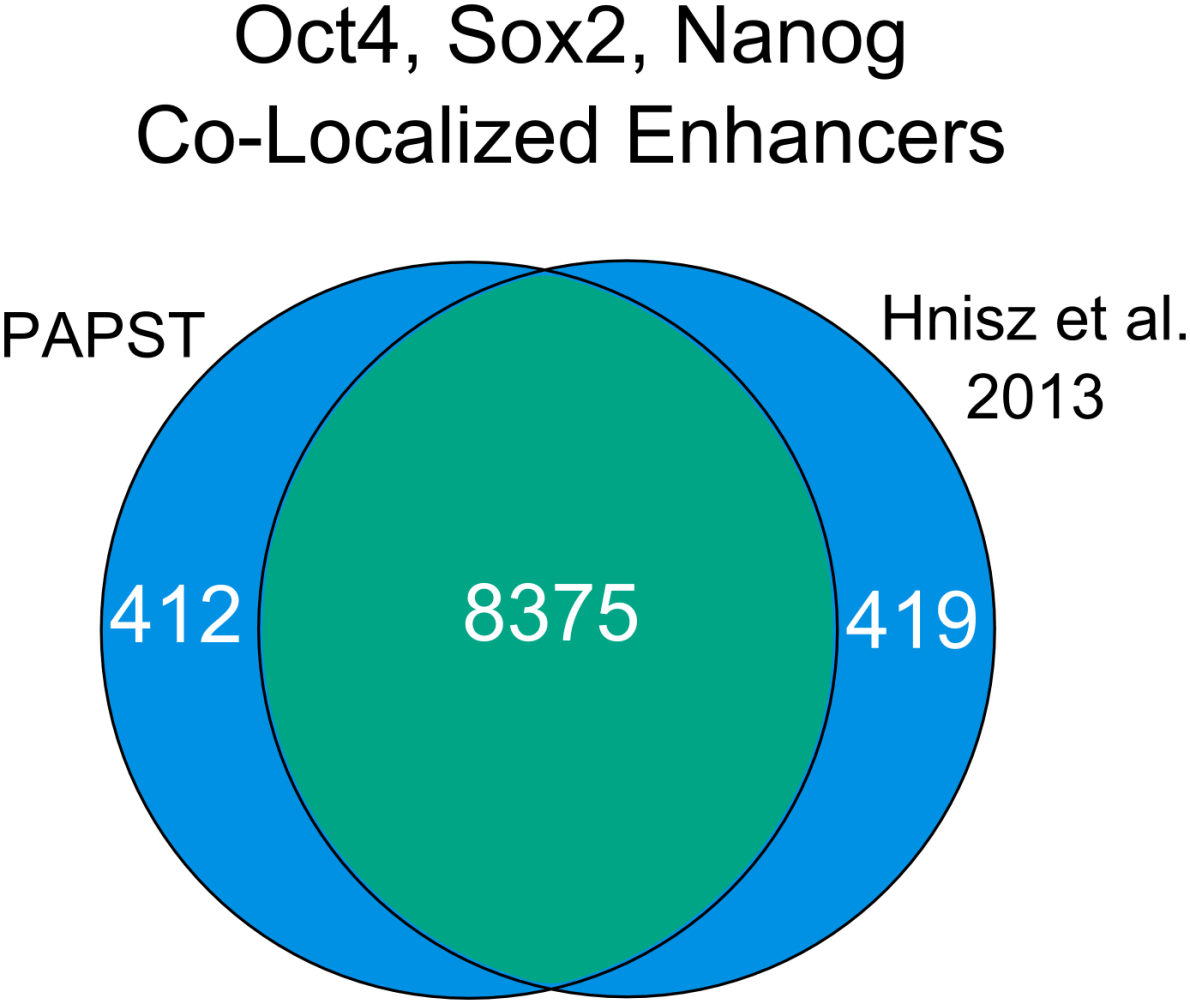
Comparison of PAPST Derived Enhancers to the Literature. Comparison of PAPST identified ESC enhancers with published ESC enhancers [16] shows that PAPST identified over 95% of those found by Hnisz et al. The enhancers were identified through the calculation of genomic regions co-localized by Oct4, Sox2, and Nanog.

Efficient co-localization analysis, both peak-centric and gene-centric, is the key feature of PAPST. ESC enhancers are characterized by the co-occupancy of master transcription factors Oct4, Sox2, and Nanog [17]. Young’s lab has recently used this co-occupancy of the master TFs as a main criterion to identify 8794 enhancers including 231 super enhancers [16]. We applied PAPST, using peak-centric analysis, to the same mouse ESC ChIP-Seq data (after BOWTIE mapping and MACS peak calling) and identified 10110 regions that are co-localized by the three master TFs. After merging close-by regions as previously described [16], a total of 8787 enhancer regions were identified by PAPST, which overlap 95% and 99%, respectively, of the 8563 typical enhancers and 231 super enhancers reported by the paper (Fig 2). In general, given a set of genomic coordinates such as those defined by the binding peaks of a transcription factor, PAPST can be used to quickly identify unlimited number of co-localized transcription factors and/or histone modifications, or any other defined genomic regions of interest.

PAPST can also perform customizable (user defined promoter, gene body, and extended gene body) gene-centric co-localization analysis to screen for genes with a specific occupancy profile of TFs and/or EMs. For example, we have applied PAPST to identifying 1182 non-redundant ESC genes with co-promoter-occupancy of Oct4, Sox2, Nanog, and H3K27Ac (S4 Table). The gene-centric co-localization analysis can be particularly useful to address specific questions related to gene regulation (see the tutorials for more examples).

#### Analysis of peak intensity within the defined genomic regions of super enhancers vs typical enhancers

**Fig 3:**
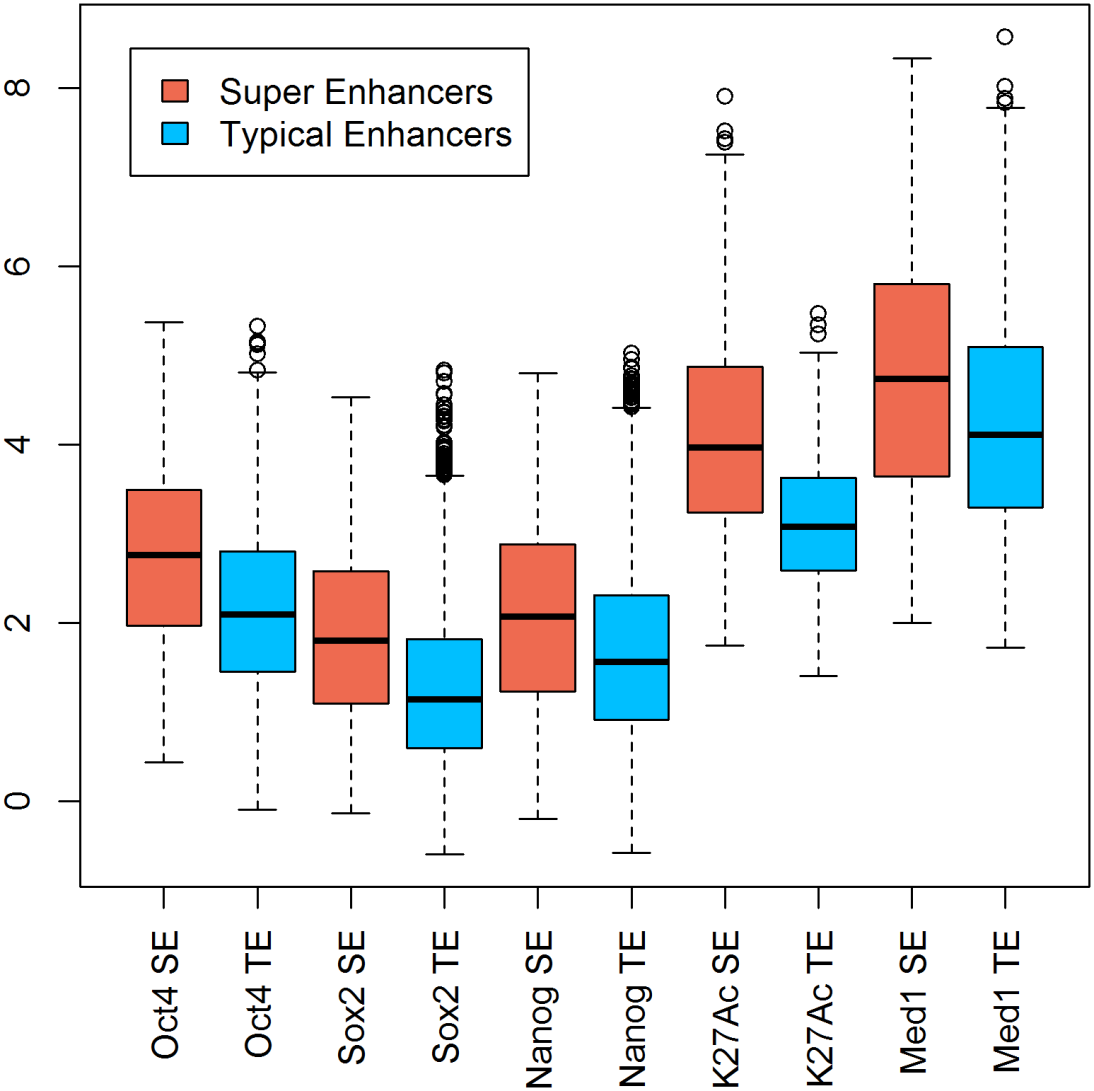
Signal Distribution of Key Transcription Factors in Super Enhancers and Typical Enhancers. Quantitative applications of PAPST compared peak signals within super-enhancers (SEs) to those within typical enhancers (TEs) of five factors showing significantly stronger signal in SEs. Red: peak signal distribution within super-enhancers. Green: peak signal distribution within typical enhancers. Peak signals are expressed as normalized read counts. All comparisons are significant with p < 1e-10 (Welch's t-test).

**Fig 4:**
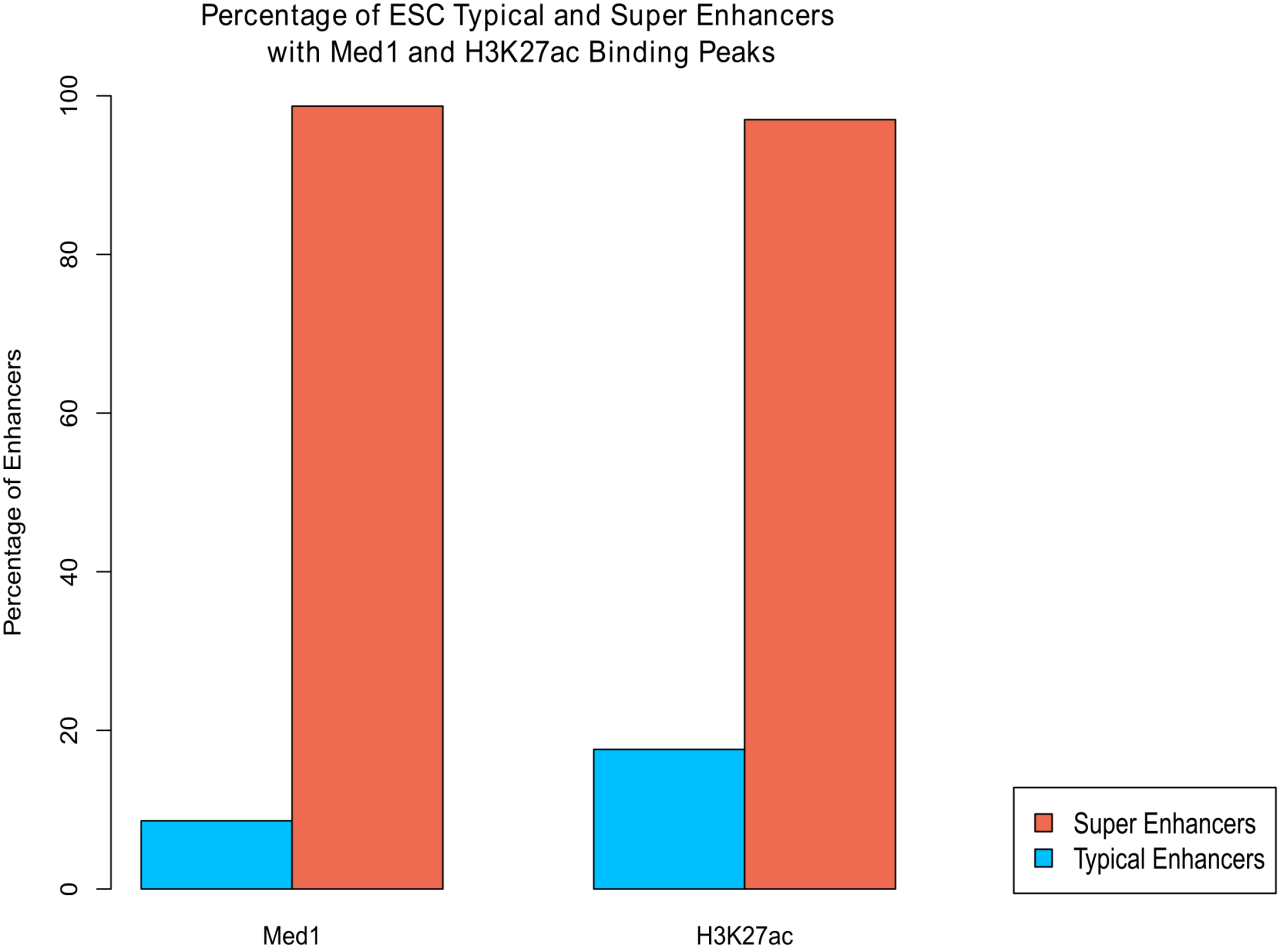
Comparison of Mediator (Med1) and H3K27ac occupancy in Super Enhancers and Typical Enhancers. PAPST quickly identified that significantly more SEs are occupied by Med1 and H3K27Ac than TEs. Quickly answering questions of this nature make PAPST ideal for exploratory research.

PAPST can generate quantitative data for extended co-localization analysis. ESC super enhancers have been shown to have higher levels of active enhancer epigenetic mark H3K27Ac and binding of key TFs as compared to typical enhancers [16]. We used PAPST to perform total tags based peak signal normalization of ChIP-Seq peaks for Oct4, Sox2, Nanog, Mediator (Med1), and H3K27Ac. Next PAPST was used to generate normalized read signals for these factor’s peaks in super enhancer regions and in typical enhancer regions respectively. The comparative results are shown in Fig 3, which indicate significantly higher levels of these key factors in the super enhancer associated peaks than those associated with the typical enhancers (p-values are: Oct4 2.26E-33, Sox2 1.96E-30, Nanog 1.30E-19, H3K27Ac 1.78E-32, and Med1 4.17E-12 using Welch's t-test). We also used PAPST to quickly generate the co-localization data showing a significantly higher percentage of super enhancers are occupied by H3K27Ac and Med1 compared to typical enhancers (Fig 4). In these rapid applications of PAPST (see the Performance and Usability section above for the timings), co-localized peaks are not only easily identified, but they can also be investigated quantitatively.

#### Assignment of super enhancers to key ESC genes by proximity

**Fig 5:**
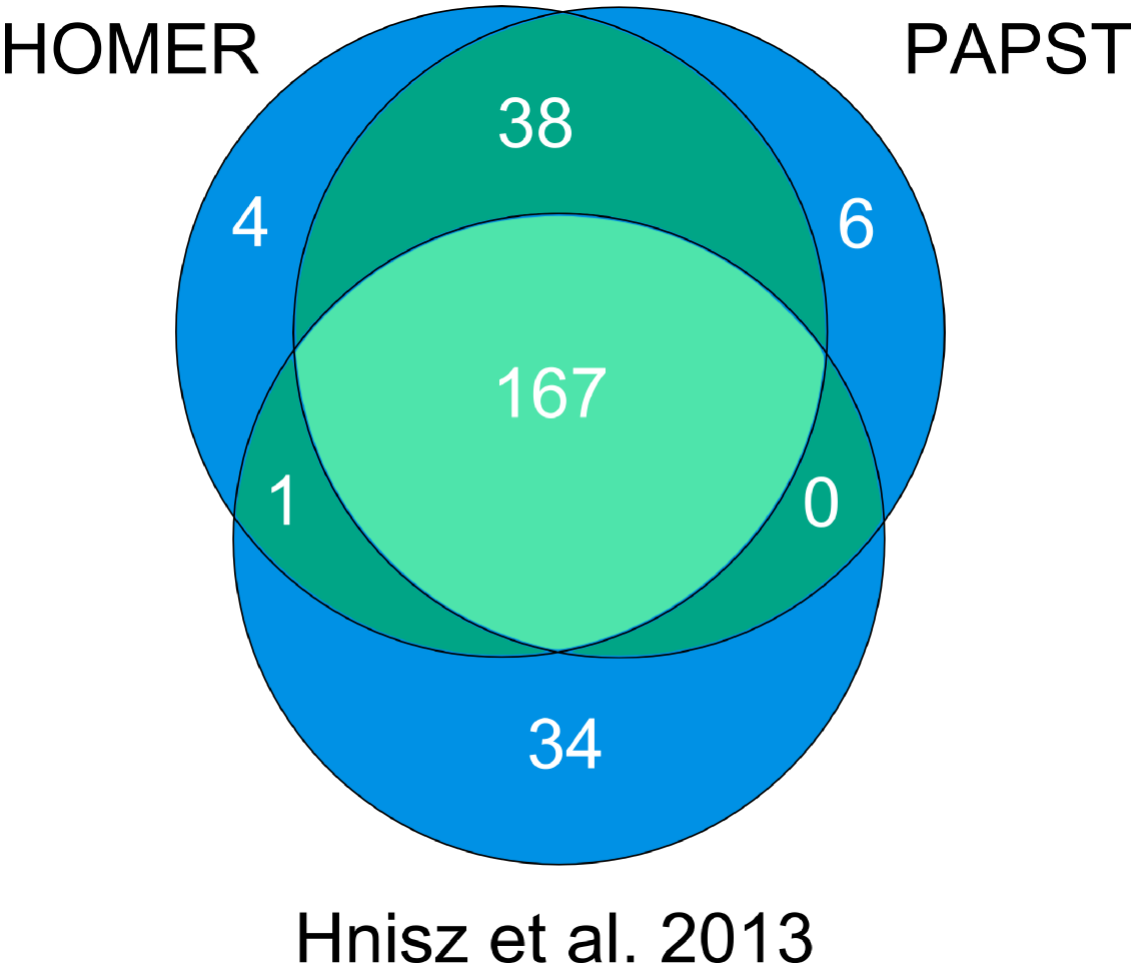
PAPST Gene Assignment of Super Enhancers Compared to HOMER and the Literature. PAPST was compared to two others gene assignments for super enhancer regions. PAPST and HOMER are 98% consistent with each other. The discrepancy with the Hnisz et al. [16] assignment is most likely due to the different versions of RefGene used (mostly new non-coding gene annotations).

PAPST can quickly assign a set of genomic regions such as ChIP-Seq peaks to genes by proximity to their transcription start site (TSS). Using PAPST, we assigned ESC super enhancers to their closest genes. The complete assignment results are presented in the supplemental materials (S5 Table). Importantly, among these PAPST assigned super enhancer associated genes are the 14 key ESC cell identity genes (Oct4, Sox2, NANOG, Tet1, Tet2, Dppa5a, n-Myc, Tbx3, Utf1, Esrrb, Prdm14, Klf4, Sall4, and Zfp42) that were also identified as the super enhancer associated genes in the original paper [16]. We have also assigned these super enhancers to genes using HOMER [13]. A comparison of the gene assignment results obtained with PAPST and the other two methods is given in Fig 5, and shows that PAPST and HOMER assigned these super enhancers to the same set of genes with an agreement of greater than 98%. The difference between the PAPST and HOMER assigned genes and those assigned by the original paper is most likely due to the specific version of RefGene used in the analysis. The minor differences between PAPST and HOMER assigned genes reflect the specific assignment algorithm’s implementation details and likely represent differences in the transcript sort order of genes with multiple isoforms.

#### Peak overlap calculation and clustering reveals known ESC transcription factor relationships

**Fig 6:**
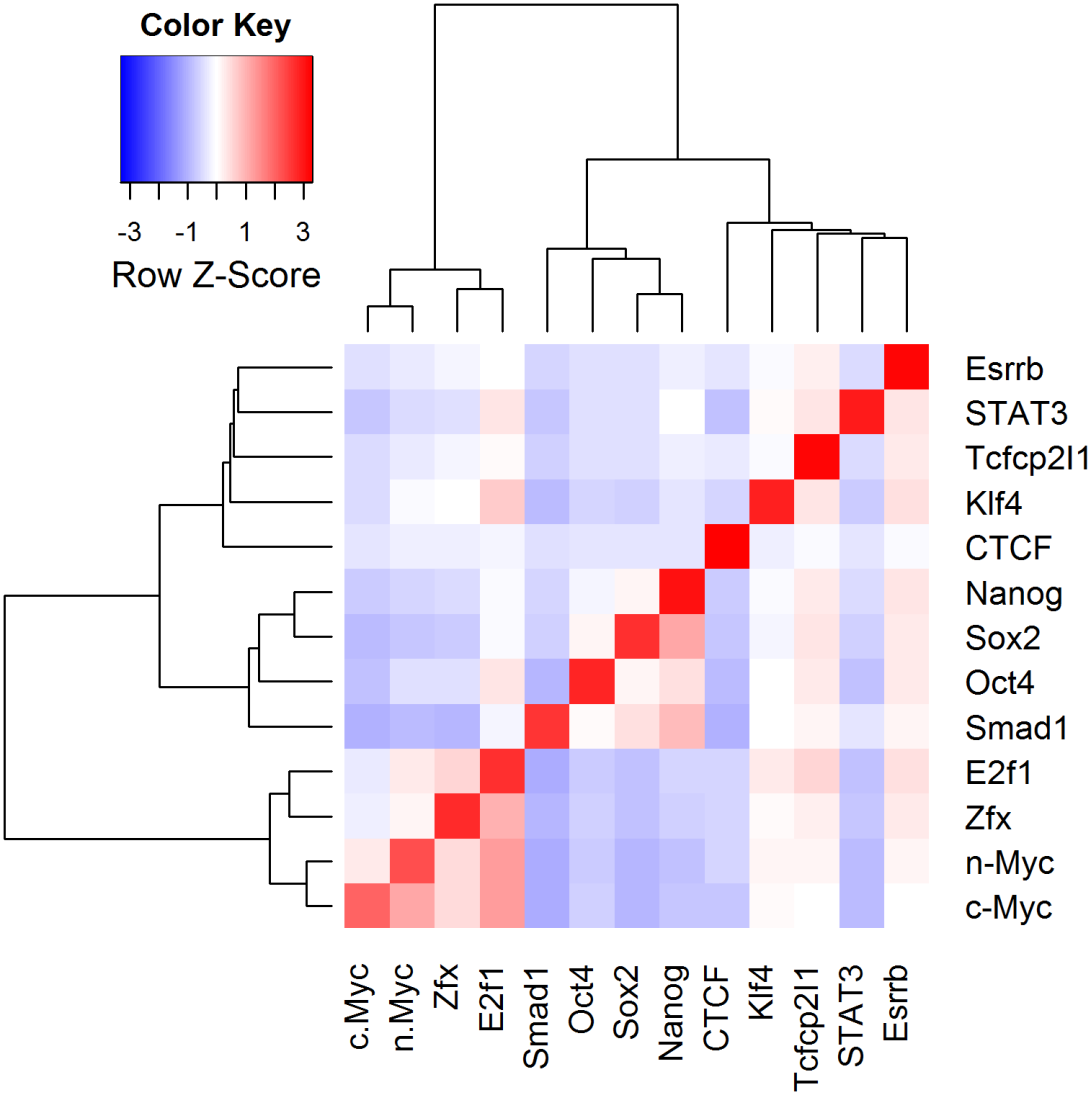
Overlap Based Clustering of 13 ESC Transcription Factors. Using PAPST’s symmetric overlap algorithm, 13 ESC Transcription were clustered. The heatmap shows the association of the 3 key reprogramming factors Oct4, Sox2, and Nanog. These groupings closely match those of Chen et al. [17].

PAPST has implemented a novel algorithm for symmetric peak overlap calculation, determining the total number of overlapping peaks for all possible pairwise comparisons of TFs and EMs included in an analysis, with one single mouse click (see Methods for details). The symmetric overlapping relationships table may then be used for binding profile based clustering. We have used PAPST to calculate the overlapping peaks of 78 pairwise comparisons among 13 ESC transcription factors (S6 Table), using another ChIP-Seq data set publicly available [17]. The results were used to cluster these factors (see S1 Appendix for details). The clustering patterns obtained with PAPST generated data (Fig 6) are virtually identical to those published, which were created with a different set of mostly command-line based tools and algorithms [17].

## Discussion

We have demonstrated PAPST’s main features by applying it to two publicly available ChIP-Seq data sets from embryonic stem cell research [16, 17]. Our results, which were obtained with PAPST on the scale of seconds, are highly consistent with those presented in the original research publications, validating the specific computational algorithms implemented in PAPST.

PAPST has been developed based on our extensive experience in ChIP-Seq data analysis [18–21], which has helped us to realize the need for an easy to use yet powerful software tool for post peak-calling ChIP-Seq data analysis that is more accessible to the researchers in the field, including bench scientists. And as such, PAPST has been designed to offer a focused set of powerful and convenient features that are very easy to use for researchers with or without computational expertise.

Two of the most powerful and unique features of PAPST are: 1) simultaneous co-localization analysis of a large number of TFs and EMs (limited only by a computer’s memory), and 2) the extremely fast return of the analysis results on the scale of seconds. A co-localization calculation of 50 factors took only 1.39 seconds and 1.54 seconds for peak-centric analysis and gene-centric analysis, respectively. As most functions of PAPST may be completed basically as fast as one can click the mouse (Table 1), and the analysis results can be easily reformatted into an input file within the package for a new round of analysis, PAPST can serve as a powerful and efficient tool for data-driven exploratory research. This is particularly true when combined with the flexible feature of PAPST that allows easy and highly customizable parameter adjustment.

PAPST offers both gene centric and peak centric features in a single package, capable of analyzing ChIP-Seq peaks both on gene-defined genomic regions (promoters, exons, introns, gene bodies) and on peak-defined genomic regions, making it a true genome-wide data analysis platform. Its peak-centric feature can be creatively and easily extended to include the co-localization analysis of TFs and EMs on any set of user-defined genomic regions of interest, such as the ENCODE determined locations of regulatory sequences [22], regions of evolutionary conservation [23], or non-coding RNAs [24]. PAPST can thus be used to address both types of general questions in ChIP-Seq data analysis: a) which genes have a specific co-localization profile of TFs and EMs? and b) which genomic locations of interest are co-localized by a set of TFs and EMs? The interesting genes identified by PAPST can be further analyzed in combination with other data types such as those from RNA-Seq or subject to a pathway analysis. Those co-localized regions identified by PAPST can either be used for extended co-localization analysis or for further downstream analysis such as motif and composition analysis. In addition, as the core analysis performed by PAPST is genomic interval based, it may also potentially find its applications in any genome data analysis that involves genomic interval based calculations.

Finally, PAPST can also generate quantitative data matrices in the form of normalized peak reads within regions defined by genes or peaks, making it possible to study epigenetic and regulatory data with other quantitative statistical methods such as PCA, K-means and hierarchical clustering, and statistical learning based modeling.

## Materials and Methods

### Data, read mapping, and peak calling

The raw data was collected from NCBI GEO using accessions GSE44288 [16] and GSE11431 [17]. Reads were mapped with Bowtie 0.12.8 [2] against mouse genome mm9. For the clustering, mm9 mapping data from the original paper were lifted to mm10 using liftOver from UCSC Genome Browser [25]. Peaks were called with MACS 1.4.2 [8]. The specific parameters used with Bowtie and MACS were chosen as close to those used in the original papers as practically possible. The boxplots and the clustering were made with R. Complete details are available in S1 Appendix.

### Design and Implementation

PAPST is built on Java 1.7 (available at http://java.com/en/download/). It operates on the statistically significant peaks called from ChIP-Seq experiments. Any BED-like file is accepted by PAPST. An in-app input wizard allows users to create parsers for custom formatted files as long as the chromosome, start and end location, and tag count or score value are present. Gene and exon information is loaded into PAPST using RefGene files, and PAPST supports all genomes available from the UCSC Genome Browser (GB). The specific RefGene data used in this study were downloaded from GB site on October 13, 2014.

Internally, sets of peaks are represented by hashmaps of nested containment lists (NCL) [26]. Given a chromosome, a hashmap returns the NCL in constant time. The NCL answers range intersection queries in O(log n) time. For PAPST, this data structure makes operating on data from a large number of experiments fast and efficient.

### Peak Overlap Calculation and Clustering

**Fig 7:**
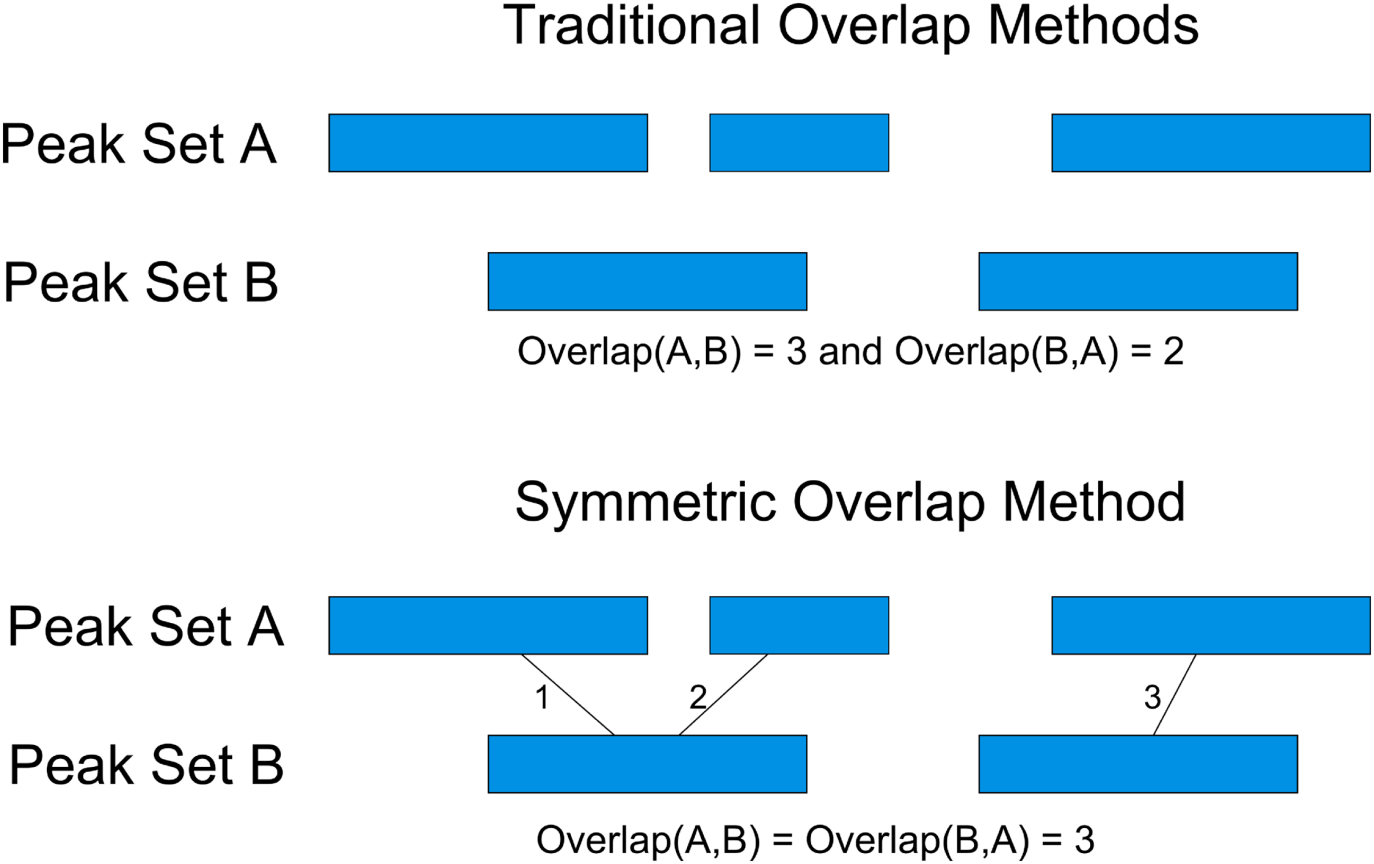
PAPST’s Symmetric Overlap of Peak Regions. PAPST uses a novel overlap algorithm which counts the number of edges drawn between overlapping peaks. This method ensures that two sets, A and B, produce a symmetric overlap relationship where Overlap(A,B) = Overlap(B,A).

Most ChIP-Seq analysis programs calculate the overlap of peak regions in a non-symmetric way. Problems arise when a single peak region from one experiment overlaps multiple peaks in another experiment. For applications such as clustering, having a symmetric overlap relationship is desirable. PAPST uses a novel overlap algorithm that ensures the binary relationship between two peaks sets is symmetric (i.e. Overlap(A,B) = Overlap(B,A)). The algorithm accomplishes this by counting the number of edges that would be drawn to connect a peak with its overlapping peaks in the opposite set (Fig 7). When applied to multiple peak sets, PAPST generates a symmetric matrix of overlap values that is suitable for clustering. R was used to cluster 13 ChIP-Seq experiments based on their binding profiles using Ward’s method. Before clustering, the data were normalized to have a mean of zero and unit variance. Details are available in the supplementary materials and tutorials.

### Special term definitions

**Gene-centric**: ChIP-Seq data analysis on genomic regions defined by genes, such as promoters, exons, introns, and gene bodies. The analysis result usually is a set of genes with shared ChIP-Seq features, such as genes with their promoters co-occupied by two transcription factors

**Peak-centric**: ChIP-Seq data analysis on genomic regions defined by a set of ChIP-Seq peaks. The analysis result usually is a set of genomic regions with shared ChIP-Seq features, such as those regions co-occupied by a transcription factor and a histone modification

**Typical enhancer**: normal enhancer

**Super enhancer**: a cluster of adjacent typical enhancers

## Supporting Information

S1 Appendix The appendix contains supplementary methods that describe data collection, generation, preprocessing, and normalization methods. (DOC)

S1 Table Assignment of 6912 Oct4 ChIP-Seq Peaks to the nearest TSS. The data presented in this table provides an example of the gene assignment function of PAPST.
(CSV)

S2 Table Search Results for Genes Bound by Oct4 in the 2000 bp Upstream Promoter. This file contains the table for all unique genes (isoforms excluded) that have a significant peak located in the promoter regions 2000 up stream of the TSS.
(CSV)

S3 Table Search Results for Genes Bound by both Oct4 and K4me3 in the 2000 bp Upstream Promoter. This file contains the table for all unique genes (isoforms excluded) that have a significant peak for both Oct4 and K4me3 located in the promoter regions 2000 up stream of the TSS.
(CSV)

S4 Table Non-redundant ESC Genes with Promoter Co-occupancy of Oct4, Sox2, Nanog, and H3K27ac. PAPST searched for gene promoters (+/- 5000 bp from TSS) that contained a binding profile that included all 3 transcription factors and the H3K27ac epigenetic mark. This table contains the exhaustive list of identified target genes.
(XLSX)

S5 Table Assignment of Super Enhancers to their Nearest Gene. Using PAPST’s gene assignment feature, all super enhancer regions were assigned to the nearest TSS. This table contains all the assigned genes’ accessions, both the enhancer and gene coordinates and the distance from the enhancer to the TSS.
(XLSX)

S6 Table Pairwise overlapping matrix generated by PAPST for 13 ESC transcription factors. PAPST was used to generate the all-pairs overlap data. Data generated from overlap comparisons in PAPST is symmetric making it suitable for clustering as well as other multi-dimensional analyses.
(CSV)
