# Supplementary material for: PAPST, a User Friendly and Powerful Java Platform for ChIP-Seq Peak Co-Localization Analysis and Beyond
Source: PLoS One. 2015 May 13;10(5):e0127285. doi: 10.1371/journal.pone.0127285 (PMC4430287; doi:10.1371/journal.pone.0127285)
Supplement: S1 Appendix — (DOC) [file pone.0127285.s001.doc]

# S1 Appendix

### 1. Embryonic stem cell data and PAPST Tutorial.

An in-depth tutorial and user guide is provided online at <https://github.com/paulbible/papst>. It is recommended that users start with this tutorial to learn the basic and advanced features of PAPST. The tutorial features step-by-step instructions with images for both Mac and Windows users. The tutorial and user guide target researchers without any bioinformatics expertise. The only system requirement is Java 1.7 or later. The latest version of Java can be downloaded at <https://java.com/en/download>.

The tutorial features mouse transcription factor ChIP-Seq data from and histone modification ChIP-Seq data from . Raw sequencing data for these experiments were taken from NCBI Geo using accessions GSE11431 (TF ChIP-seq) and GSE12241 (Histone modifications). These sequence data are referred to collectively as the ESC dataset. Sequence data were converted from mm8 to mm10 using liftOver from UCSC Genome Browser . Significant peaks were called with MACS 1.4.2 after the protocol by Feng et al. . Since no control samples were available for these NCBI GEO series, MACS was called using the recommended settings for p-values (1e-5) for a single sample. FDR values from MACS models are only calculated for samples with controls. For histone modifications, the “--nomodel” parameter was used to accommodate the broader peaks derived from histone modifications. These data offer a rich set of interesting TF and histone binding data that the user can quickly explore using PAPST.

### 2. Clustering of Chen et al. 2008 ESC data

To produce the clustering of the ESC transcription factors provided in the Fig. 5 of the text, the following procedure was used.

1. Using the ESC dataset, we loaded Esrrb, Stat3, Tcfcp2l, Klf4, CTCF, Nanog, Sox2, Oct4, Smad1, E2f1, Zfx, n-Myc, and c-Myc peaks into PAPST (available in the tutorial download).
2. With these peak sets selected, we clicked the ‘Compare’ button in PAPST to create the symmetric overlap matrix for these TFs.
3. We exported the overlap matrix using the ‘Export Table’ button on the ‘Data’ tab to export the matrix to a comma separated values (csv) file named ‘results_cell.csv’.
4. The heatmap was created using the R Programming Language and the gplots package using the following commands:

library(gplots)

pearson <- function(x){as.dist((1-cor(t(x)))/2)}

hfun <- function(x){hclust(pearson(scale(d)),method="ward")}

d <- read.table("results_cell.csv",sep=",",header=T,row.names=1)

heatmap.2(as.matrix(d),col=bluered(100),scale="row",density="none",trace="none",\

hclustfun=hfun,mar=c(10,10))

### 3. Normalization

An optional feature of PAPST allows peaks to be normalized for different sequence depths. Using the tag count provided for each peak and a user provided total tag count, PAPST will adjust each individual peak value to reflect the relative tag count per million reads. This formula is given below.


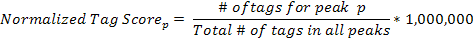


This feature will normalize each peak value to allow comparisons between multiple data sets with different sequencing depths. PAPST allows the user to customize the normalization value (default 1 million) in the above formula. If the peak file’s values have already been normalized then this feature can be ignored. This gives the user the freedom to use other normalization methods before inputting peaks into PAPST. More information on normalization can be found in the tutorial and user guide at <https://github.com/paulbible/papst> under the tutorials folder.

[1] X. Chen, H. Xu, P. Yuan, F. Fang, M. Huss, V. B. Vega*, et al.*, "Integration of external signaling pathways with the core transcriptional network in embryonic stem cells," *Cell,* vol. 133, pp. 1106-17, Jun 13 2008.

[2] T. S. Mikkelsen, M. Ku, D. B. Jaffe, B. Issac, E. Lieberman, G. Giannoukos*, et al.*, "Genome-wide maps of chromatin state in pluripotent and lineage-committed cells," *Nature,* vol. 448, pp. 553-60, Aug 2 2007.

[3] T. Barrett, S. E. Wilhite, P. Ledoux, C. Evangelista, I. F. Kim, M. Tomashevsky*, et al.*, "NCBI GEO: archive for functional genomics data sets--update," *Nucleic Acids Res,* vol. 41, pp. D991-5, Jan 2013.

[4] A. S. Hinrichs, D. Karolchik, R. Baertsch, G. P. Barber, G. Bejerano, H. Clawson*, et al.*, "The UCSC Genome Browser Database: update 2006," *Nucleic Acids Res,* vol. 34, pp. D590-8, Jan 1 2006.

[5] Y. Zhang, T. Liu, C. A. Meyer, J. Eeckhoute, D. S. Johnson, B. E. Bernstein*, et al.*, "Model-based analysis of ChIP-Seq (MACS)," *Genome Biol,* vol. 9, p. R137, 2008.

[6] J. Feng, T. Liu, B. Qin, Y. Zhang, and X. S. Liu, "Identifying ChIP-seq enrichment using MACS," *Nat Protoc,* vol. 7, pp. 1728-40, Sep 2012.

[7] R Core Team, "R: A Language and Environment for Statistical Computing," ed. Vienna, Austria, 2013.

[8] G. R. Warnes, B. Bolker, L. Bonebakker, R. Gentleman, W. H. A. Liaw, T. Lumley*, et al.*, "gplots: Various R programming tools for plotting data," ed, 2014.
